# "Healthy Eating - Healthy Action": evaluating New Zealand's obesity prevention strategy

**DOI:** 10.1186/1471-2458-9-452

**Published:** 2009-12-06

**Authors:** Rachael M McLean, Janet A Hoek, Sue Buckley, Bronwyn Croxson, Jacqueline Cumming, Terry H Ehau, Ausaga Fa'asalele Tanuvasa, Margaret Johnston, Jim I Mann, Grant Schofield

**Affiliations:** 1 Department of Medical and Surgical Sciences, University of Otago, New Zealand; 2 Health Services Research Centre, School of Government, Victoria University of Wellington, New Zealand; 3 Native Consultancy Ltd, Maketu, Te Puke, New Zealand; 4 School of Sport & Recreation, Centre for Physical Activity and Nutrition, Auckland University of Technology, New Zealand

## Abstract

**Background:**

New Zealand rates of obesity and overweight have increased since the 1980s, particularly among indigenous Māori people, Pacific people and those living in areas of high deprivation. New Zealand's response to the obesity epidemic has been The *Healthy Eating-Healthy Action: Oranga Kai - Oranga Pumau (HEHA) Strategy *('the Strategy'), launched in 2003. Because the HEHA Strategy explicitly recognises the importance of evaluation and the need to create an evidence base to support future initiatives, the Ministry of Health has commissioned a Consortium of researchers to evaluate the Strategy as a whole.

**Methods:**

This paper discusses the Consortium's approach to evaluating the HEHA Strategy. It includes an outline of the conceptual framework underpinning the evaluation, and describes the critical components of the evaluation which are: judging to what extent stakeholders were engaged in the process of the strategy implementation and to what extent their feedback was incorporated in to future iterations of the Strategy (continuous improvement), to what extent the programmes, policies, and initiatives implemented span the target populations and priority areas, whether there have been any population changes in nutrition and/or physical activity outcomes or behaviours relating to those outcomes, and to what extent HEHA Strategy and spending can be considered value for money.

**Discussion:**

This paper outlines our approach to evaluating a complex national health promotion strategy. Not only does the Evaluation have the potential to identify interventions that could be adopted internationally, but also the development of the Evaluation design can inform other complex evaluations.

## Background

New Zealand rates of obesity and overweight have increased since the 1980s, particularly among indigenous Māori people, Pacific people and those living in areas of high deprivation[1] The 2006/7 New Zealand Health Survey reported that 29% of New Zealand children aged 2-14 years and 63% of New Zealand adults were classified as overweight or obese by World Health Organization guidelines (see table 1)[2] This is consistent with international trends which have been called a worldwide epidemic of obesity[3,4]

**Table 1:** Prevalence of Obesity by ethnicity 2006/07 New Zealand Health Survey (displayed as Prevalence % (95% CI) [2]

		Underweight	Normal range	Overweight	Obese (all classes)
**Children** **2-14 years**	Total NZ	2.9 (2.2, 3.6)	67.9 (66.2,69.6)	20.9 (19.2,22.6)	8.3 (7.4, 9.3)
	
	European/Other				5.5 (4.3, 6.7)
	
	Māori				11.8 (9.9, 13.7)
	
	Pacific				23.3 (19.7, 26.8)
	
	Asian				5.9 (3.5, 8.3)

**Adults**	Total NZ	1.3 (1.0, 1.6)	36.1 (36.0, 37.1)	36.2 (35.2, 37.1)	26.5 (25.5 - 27.5)
	
	European/Other				24.3 (23.1, 25.5)
	
	Māori				41.7 (39.8 - 43.7)
	
	Pacific				63.7 (60.0, 67.5)
	
	Asian				11.0 (9.0, 13.0)

New Zealand's response to the obesity epidemic has been The *Healthy Eating-Healthy Action: Oranga Kai - Oranga Pumau (HEHA) Strategy *('the Strategy'), launched in 2003 to address growing concerns over poor eating habits, lack of physical activity, and the associated prevalence of obesity and increased risk of adverse health outcomes that result. The Strategy's framework recognises the importance of reducing health inequalities and the Treaty of Waitangi, a treaty signed by Māori and the Crown in 1840, and considered to be New Zealand's founding document. The principles of the Treaty of Waitangi of partnership, participation and protection are enshrined in much of New Zealand's health legislation.

**Figure 1:**
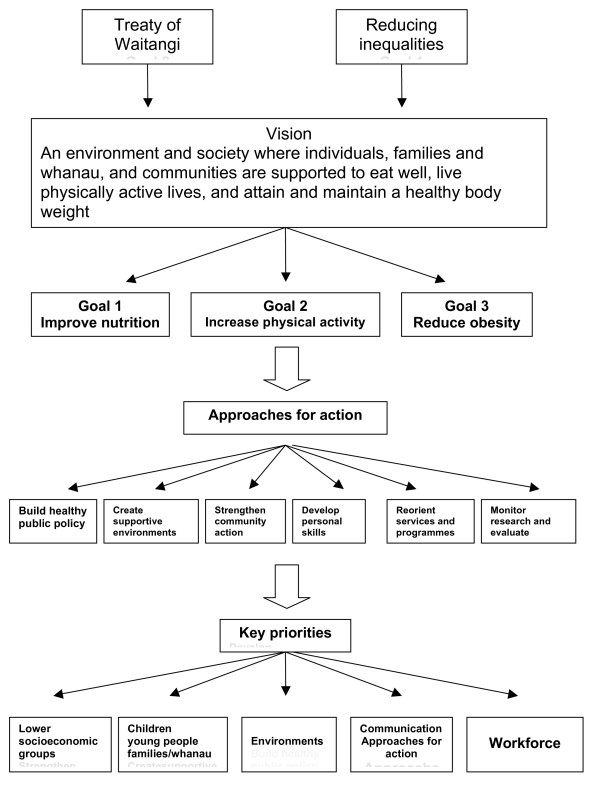
**The HEHA Framework **[6].

The Strategy articulates a vision of: "an environment and society where individuals, families and whānau, and communities are supported to eat well, live physically active lives, and attain and maintain a healthy body weight" [[5], p15] It is supported by a HEHA Implementation Plan for 2004 - 2010, which draws on the health promotion model set out in the World Health Organization's Ottawa Charter for health promotion[6] This Plan defines proposed actions and identifies target priority groups, which include Māori and Pacific peoples, as well as lower socioeconomic groups, and children, young people and their families/whānau[7] (see figure 1)

Because the HEHA Strategy explicitly recognises the importance of evaluation and the need to create an evidence base to support future initiatives, the Ministry of Health has commissioned a consortium of researchers to evaluate the Strategy as a whole. Internationally, while there is increasing evidence of effective interventions [8,9], and systematic reviews of programmes to improve nutrition, increase physical activity and reduce obesity [10-13], evidence relating to national strategies that have intervened at a population level to reduce obesity is lacking [11,14].

"[T]here are as yet no models to follow because no country has yet developed and implemented a coherent programme of action to prevent further weight gain in the population and to manage its current obesity burden." [[15], p24]

We are unaware of any documented comprehensive approach detailed to evaluate both implementation and outcomes of a national, government-lead strategy to reduce obesity. The framework and methods developed for New Zealand offer some insight into this process, and how a national level evaluation might work.

The critical components are judging:

1. To what extent stakeholders were engaged in the process of the strategy implementation and to what extent their feedback was incorporated in to future iterations of the Strategy (continuous improvement)

2. To what extent the programmes, policies, and initiatives implemented span the target populations and priority areas

3. Whether there have been any population changes in nutrition and/or physical activity outcomes or behaviours relating to those outcomes

4. How much was spent on different programmes, and to what extent this spending can be considered value for money.

This paper discusses the Consortium's approach to evaluating the HEHA Strategy. This research has been approved by the New Zealand Multi-region Health and Disability Ethics Committee and the University of Otago Ngai Tahu Research Consultation Committee.

## Methods

### Conceptual Framework

The HEHA Strategy takes an ecological approach and recognises that environmental and socio-demographic factors influence nutrition, physical activity, and obesity. In addition, the HEHA framework draws on the Treaty of Waitangi, the Ottawa Charter [6] and two key Ministry of Health Strategies: *He Korowai Oranga-Maori Health Strategy *[16] the Pacific Health and Disability Action Plan[17] Because the Strategy recognizes multiple stakeholders, it has both horizontal and vertical complexity [18] and involves government and non-government organisations, as well as community and industry groups. In addition, the Strategy's effects are intended to be experienced at all levels, including organisations, agencies, communities, families/whānau and individuals. To achieve these outcomes, the Strategy must co-ordinate activities between disparate groups, which themselves have varying goals and objectives, while also meeting the needs of different stakeholders. In particular, the emphasis on Māori and Pacific models of health means the Strategy must recognise multiple cultural models.

The Strategy is dynamic and thus may be influenced by changing political and social contexts. The complexity of the environment, the program and the problem is reflected in the complexity of the evaluation. As noted by other evaluators of complex programmes, they: "cannot be captured within one overarching theory" [[19], p274] A number of different theories are therefore being used to guide the evaluation, as described in the following sections.

#### Priority Groups: Māori and Pacific

The evaluation recognises the importance of the Treaty of Waitangi to New Zealand policymaking and the Treaty principles of partnership, protection and participation are reflected in the evaluation methodology. More specifically, the evaluation recognises the importance of Māori values, needs and aspirations to policy processes and interventions. [[20], p56] The HEHA Strategy expects Māori to play an important role in designing and delivering HEHA initiatives, but it also locates responsibility for improving Māori nutrition and physical activity levels and support services with mainstream providers [5,4-10]. Evaluation of the HEHA Strategy thus requires a Māori lens that can examine how Māori-specific and mainstream initiatives have involved Māori and improved Māori health outcomes.

Māori researchers within the consortium have used a number of Māori models of health (including Durie's *Te Pae Mahutonga*, [21]*Hua Oranga*, [22] and *He Taura Tieke *[23]) to develop evaluation principles that acknowledge Māori development, particularly healthy lifestyles for Māori and greater social participation, and Māori autonomy, especially as this relates to priority setting and self-determination. The principles also emphasize Māori delivery of services, leadership in evaluation, and the need for integration with aligned sectors.

The HEHA Strategy also recognises the importance of reaching Pacific peoples, who are disproportionately affected by obesity-related health problems [5]. Pacific people make up 7% of New Zealand's overall population and comprise people from many Pacific Island nations (including Samoa, Tonga, Cook Islands, Fiji, Tokelau, Niue, and Tuvalu) as well as those born in New Zealand. The Pacific evaluation framework has been informed by the principles of *The Pacific Health and Disability Action Plan *(2002), particularly dignity and sacredness in the delivery of health; active participation of Pacific peoples in all levels of health and disability; Pacific leadership and communities with successful Pacific services; and Pacific people's entitlement to excellent health. Interventions affecting Pacific peoples need to recognise their holistic view of health and be sensitive to the important cultural significance that food has to them[5,20] Furthermore, the evaluation recognises that there is no generic 'Pacific community' but rather Pacific peoples who align themselves variously, using ethnic, geographic, church, family, school, age/gender, island born/New Zealand born or occupational lines.

Because the meaning of health to Pacific people varies according to each Pacific context, the evaluation will use the '*Fono Fale*' model of health. This model aligns with the principles of *The Pacific Health and Disability Action Plan *[24] and the Ottawa Charter for Health Promotion. Developed by Fuimaono Karl Pulotu-Endermann for use in New Zealand, the '*Fono Fale' *model incorporates the values of family, culture and spirituality. It uses the metaphor of a Samoan fale or house with the roof representing cultural values and beliefs and the foundation representing the family or community. The four pou or posts connect the culture and family and interact with each other, and include the following dimensions: spiritual; physical; mental and emotional, and other factors, such as gender, sexual orientation, age, social class, employment and educational status, which can affect health[25] This framework is intended to focus effort and support relationships and collaborations among the diverse Pacific stakeholders that, in turn, will provide insights into whether the HEHA Strategy has delivered improved outcomes for Pacific people.

#### Evaluation Theories

These approaches recognise that social programmes take place in diverse contexts and that the interrelationships, institutions and structures of the contexts into which a programme is introduced all shape its outcome. A realistic evaluation framework enables separation of the contexts, mechanisms and outcomes associated with a programme of change such as the HEHA Strategy. Because they investigate mechanisms for change within their contexts, realistic approaches also allow for the dynamic nature of both the Strategy itself and the contexts within which it is implemented. 'Theories of Change' explores how actions lead to desired outcomes and accommodates multiple theories across programmes; as such it is particularly suited for evaluation of complex high level programmes such as the HEHA Strategy [26]. More specifically, this approach is congruent with Māori and Pacific models, particularly the partnership model articulated in the Treaty of Waitangi, and the varied settings that will be examined during the evaluation, The research design uses multiple lines of evidence to address the evaluation aim of assessing the overall HEHA Strategy as it has affected varied stakeholders, sectors and settings.

### Evaluation of Implementation

Evaluation of the Strategy's implementation will adopt a national and district perspective. At a national level, the evaluation will explore key stakeholders' engagement with the Strategy and examine how effectively the groups established to oversee the HEHA Strategy have met their objectives. These will include representatives from non-governmental organizations (NGOs), government, industry, Māori and Pacific groups. At a local level, implementation has occurred primarily through the 21 District Health Boards (DHBs), which have employed HEHA Project Managers within their planning and funding sections, to lead and co-ordinate HEHA activities in their districts. Other district, Māori iwi (tribe), whānau (extended family) and community groups are also involved in implementation at this level. The evaluation will examine the implementation infrastructure and processes and how they relate to the Implementation Plan. It will also explore how the HEHA implementation logic (or theories of change logic) was translated into processes designed to effect change. This analysis will identify barriers and enablers to effective implementation, and will examine whether the Strategy implementation has promoted change across sectors, regions, communities, organisations, settings, whānau, families and individuals, with a particular focus on the priority populations targeted by the Strategy. Overall, this work will inform conclusions about how well the Strategy's implementation supported improvements in nutrition, physical activity rates and obesity levels.

These questions and an analysis of the type and pattern of implementation will be examined by reviewing national and district documents; and conducting key informant interviews and focus groups at national and district levels, including DHB staff as well as local stakeholders. A database of all HEHA initiatives will be compiled to identify the range and coverage of initiatives underway, and existing evaluations of major HEHA initiatives will be reviewed using a template developed by the Evaluation Consortium, largely based on the CDC *Framework for Program Evaluation in Public Health *[27].

### Supply and environmental interventions

Because the HEHA Strategy has an ecological approach that recognises the importance of environmental change in supporting healthy lifestyles, the Strategy evaluation will also examine how changes to food supply and the wider environment have supported the Strategy goals. This work will examine settings such as schools and other childcare centres, homes and communities, supermarkets and grocery stores and other food retail environments, and will evaluate how government policy, economic factors such as the markets, marketing and distribution of food, and initiatives to improve personal skills have promoted HEHA objectives [28].

### Outcomes

Although improving nutrition, increasing physical activity and reducing obesity are seen as long-term goals, measurement of physical activity and nutrition behaviours is fundamental to understanding the success of the Strategy. Without population level changes in these behaviours (in the right direction), the HEHA Strategy is likely to achieve few improvements in population health status. Examination of longer-term outcomes requires population data, particularly those that enable changes in priority groups (children and young people, Māori, Pacific people, and the socially disadvantaged) to be estimated. Because changes in nutrition and exercise will be influenced by myriad factors, the evaluation will use monitoring tools rather than experimental approaches. Nevertheless, even these approaches will only document short-term changes and the changes in behaviour required to address the HEHA objectives must also be estimated over the longer term and, ideally, would be assessed against specific targets.

To gain insights into the outcomes that may be linked or attributed to the HEHA strategy a new the Nutrition and Physical Activity Survey (NPAS) will be undertaken. The survey design is based on a rolling quarterly survey that establishes four 'panels' of respondents, each of which is interviewed annually across three years. Nationally representative existing datasets which include data on nutrition, physical activity patterns and body size will also be analysed and used to create benchmarks against which estimates from the NPAS will be compared.

A sample size of 6400 people fifteen years and older will be initially recruited to give (allowing loss to follow up over the three year period) a minimum of 1400 participants per quarter. Participants will be recruited from 600 randomly selected residential meshblocks. Meshblocks with a high index of deprivation,[29] as well as Maori and Pacific people will be oversampled to enable useful estimates of these priority groups to be calculated. The sample size calculations have been undertaken based on previous modelling for Ministry of Health nationally representative surveys using similar cluster sampling methods. This involves ensuring that a range of population level prevalences can be estimated by age and ethnicity, with design effects that enable suitable precision of the estimates for monitoring population changes in nutrition, and physical activity behaviours[2] Recruitment will be undertaken 'face to face', followed by annual computer assisted telephone interview (CATI) survey for three years. The survey instrument is made up of a range of questions from other national surveys as well as some new questions designed specifically for the evaluation. It includes questions about socio-demographic factors, vegetable and fruit consumption, consumption of foods high in fat, salt and sugar, physical activity and inactivity. As the HEHA Strategy has an emphasis on creating supportive environments to improve nutrition and increase physical activity the survey also contains questions about environmental and community enabling influences and barriers to healthy eating and physical activity behaviour. Estimates of population values will be derived by standard methods for analysing a multistage cluster sample. Descriptive analysis will then be undertaken, in order to explore changes in nutrition and physical activity behaviour as well as perceived changes in environmental influences. Comparison with earlier baseline nationally representative datasets will be made. Descriptive data will be presented by age, ethnic group, and a socioeconomic status using an index of deprivation assigned to each meshblock. Adjustment for potential confounding factors of age, ethnicity and socioeconomic status will be made for any population level data analysis. As the NPAS will survey only those 15 years and older, data pertaining to children will be gathered from other sources.

### Value-for-Money

Value-for-money analysis proceeds by linking inputs to outputs and outcomes, in order to assess the value inputs have generated. The complexity of the HEHA Strategy and the nature of the problem make this extremely difficult: evaluators need to be concerned with multiple inputs, multiple outputs and multiple outcomes, and the dynamic feedback loops between them. As in all complex situations it is different to attribute inputs to outputs - and this is compounded by the importance of factors from outside the HEHA Strategy itself, which may also influence nutrition, physical activity and obesity outcomes.

The application of formal economic evaluation tools to the analysis of complex situations is in its infancy [30] and requires creativity and innovation in information gathering; integrating qualitative analysis into the heart of trials and economic analysis; and being explicit about the assumptions that underlie estimates.

In order to address the question of whether the HEHA Strategy and its implementation has resulted in value for money three types of analysis will be undertaken: a direct analysis of value for money; a Programme Budget and Marginal Analysis (PBMA) to identify potential improvements in value for money; and an indirect analysis of expected value for money, using comparative institutional analysis. The value-for-money analysis is integrally connected with the other work streams, and so will be fully integrated with the other areas of research.

The direct analysis of value-for-money will identify and describe key components of HEHA, including overall funding levels and sources of funding; the specific initiatives which make up the Strategy at national and district levels; the key outputs from HEHA initiatives; and expected outcomes from those initiatives. This will be informed by the stocktake of initiatives; key informant interviews; document analyses (including budgets and contracts) and information derived from the review of evaluations. The links between resources, inputs, initiatives, outputs and outcomes (including the differential impact of HEHA) will be estimated and modelled using the best available information.

The second part of the economic evaluation involves a PBMA to identify potential improvements in value for money. PBMA comprises two broad types of activity: first, the compilation of a programme budget (when resources and current services are identified); and second, marginal analysis, which assesses the impact of changes in resource use[31] It is, in practice, difficult to assess existing resource allocation, let alone the impact and benefits of marginal changes, but PBMA provides a systematic framework for identifying the way resources are currently being allocated, and then considering how resources should be reallocated to achieve greater value[32] The PBMA will be undertaken as a separate exercise with key stakeholders, informed by information derived from all aspects of the Strategy evaluation.

The third part of the economic evaluation uses new institutional economics to evaluate the Strategy in terms of its incentives and constraints, the interests of different actors, and the historical evolution of relevant institutional arrangements. These arrangements include both formal (such as legislation) and informal institutions (e.g. norms), which have affected the development of the HEHA Strategy and which have, in turn, been affected by the Strategy. We intend examining the historic context, giving rise to the Strategy, including events in the New Zealand government and public sector, and in relevant international agencies during the 1990s. These pre-existing arrangements shaped and constrained the strategy and its implementation, and will have had ongoing effects insofar as there is institutional inertia.

## Discussion

The increasing prevalence of obesity is a major public health issue across the world. New Zealand has undertaken to reverse this trend by implementing a national Strategy with the goals of improving nutrition, increasing physical activity and reducing obesity. As yet, no national population-based anti-obesity strategies have been evaluated, thus the evaluation of the HEHA Strategy will inform on-going obesity reduction initiatives in New Zealand and internationally. The HEHA focus on reducing inequalities, particularly for Māori, Pacific and lower socio-economic groups, will also be of international interest as ethnic and socioeconomic inequalities affect many nations, and strategies that reduce these will be of enormous value to the wider public health community.

Particular challenges affect the evaluation of the HEHA Strategy (and similar strategies). HEHA is a complex intervention that is occurring in a complex situation that itself contains many unrelated factors likely to influence nutrition and physical activity environments and behaviours. Furthermore, the HEHA goals of improving nutrition, increasing physical activity and reducing obesity are long-term and may not be achieved within the evaluation time frame.

However, New Zealand's relatively small population and good sources of existing population data support a comprehensive assessment of potential outcomes. Furthermore, New Zealand's ethnically diverse population means the estimates will have international relevance. As a result, the HEHA Strategy Evaluation has the potential to identify interventions that could be adopted internationally, and could promote better health not only in New Zealand, but around the world.

## Abbreviations

CDC: Centres for Disease Control and Prevention; DHB: District Health Board; HEHA: Healthy Eating-Healthy Action; Oranga Kai: Oranga Pumau; NPAS: Nutrition and Physical Activity Survey; PMBA: Programme Budget Marginal Analysis.

## Competing interests

The authors declare that they have no competing interests.

## Authors' contributions

RM and JH drafted the manuscript. RM and JH contributed to the supply and environmental interventions and outcomes study design, GS contributed to the stocktake and outcomes study design. SB, JC, TE and AFT contributed to the conceptual framework. BC and JC contributed to the value for money study design. JM and SMJ contributed to the overall study design and its integration. All authors read and approved the final manuscript.

## Pre-publication history

The pre-publication history for this paper can be accessed here:

http://www.biomedcentral.com/1471-2458/9/452/prepub
